# A Comprehensive Model of Factors Associated With Subjective Perceptions of “Living Well” With Dementia: Findings From the IDEAL Study

**DOI:** 10.1097/WAD.0000000000000286

**Published:** 2018-12-05

**Authors:** Linda Clare, Yu-Tzu Wu, Ian R. Jones, Christina R. Victor, Sharon M. Nelis, Anthony Martyr, Catherine Quinn, Rachael Litherland, James A. Pickett, John V. Hindle, Roy W. Jones, Martin Knapp, Michael D. Kopelman, Robin G. Morris, Jennifer M. Rusted, Jeanette M. Thom, Ruth A. Lamont, Catherine Henderson, Isla Rippon, Alexandra Hillman, Fiona E. Matthews

**Affiliations:** * Centre for Research in Ageing and Cognitive Health (REACH), School of Psychology; ‡ Wellcome Centre for Cultures and Environments of Health, University of Exeter; † PenCLAHRC, University of Exeter Medical School; ¶ Innovations in Dementia, Exeter; § Wales Institute for Social and Economic Research, Data and Methods, Cardiff University, Cardiff; ∥ College of Health and Life Sciences, Brunel University London; # Alzheimer’s Society; §§ Personal Social Services Research Unit, London School of Economics and Political Science; Departments of ∥∥ Psychological Medicine; ¶¶ Psychology, King’s College London, Institute of Psychiatry, Psychology and Neuroscience, London; ** Department of Care for the Elderly, Betsi Cadwaladr University Health Board, Llandudno; †† School of Psychology, Bangor University, Bangor; ‡‡ RICE (The Research Institute for the Care of Older People), Bath; ## School of Psychology, University of Sussex, Brighton; ††† Institute for Health and Society, Newcastle University, Newcastle upon Tyne, UK; *** School of Medical Sciences, University of New South Wales, Sydney, Australia

**Keywords:** quality of life, satisfaction with life, well-being, Alzheimer

## Abstract

**Introduction:**

We aimed to better understand what predicts the capability to “live well” with dementia by identifying the relative contribution of life domains associated with the subjective experience of living well.

**Methods:**

We analyzed data from 1547 individuals with mild-to-moderate dementia in the IDEAL cohort. We generated a “living well” latent factor from measures of quality of life, satisfaction with life, and well-being. We used multivariate modeling to identify variables related to living well measures and structural equation modeling to derive latent variables for 5 life domains and to examine the associations of these domains with living well.

**Results:**

All 5 domains were individually associated with living well. When modeled together, the psychological characteristics and psychological health domain was the only independent predictor of living well [effect size, 3.55; 95% confidence interval (CI): 2.93-4.17], and effect sizes were smaller for physical fitness and physical health (1.23, 95% CI: −0.10 to 2.58), social capitals, assets and resources (0.67; 95% CI: −0.04 to 1.38), managing everyday life with dementia (0.33; 95% CI: −0.06 to 0.71), and social location (0.08; 95% CI: −2.10 to 2.26).

**Discussion:**

Psychological resources, and the social, environmental, and physical factors that underpin positive psychological states, are potentially important targets for interventions and initiatives that aim to improve the experience of living with dementia.

## Abstract 2

Supplemental Digital Content is available in the text.

To live well with chronic illness and disability means experiencing “the best achievable state of health that encompasses all dimensions of physical, mental and social well-being,” reflected in “a self-perceived level of comfort, function and contentment with life.”^1^^(p32)^ The concept of living well is now frequently mentioned in policy documents and reports relating to dementia,^2^,^3^ and is used to convey the message that it is, or should be, possible to experience a subjective sense of “comfort, function and contentment with life” while living with the condition. This reflects a move from a focus on symptoms and “deficits” to a broader focus acknowledging personhood and the rights of people with dementia, enabling optimal functioning, and supporting participation and inclusion.

In the research context, the subjective experience of living well is typically equated with experiencing a good quality of life (QoL).^4^ QoL is a wide-ranging construct defined as representing “an individual’s perceptions of their position in life in the context of the culture and value systems in which they live and in relation to their goals, expectations, standards and concerns” and affected by a person’s “physical health, psychological state, level of independence, social relationships, personal beliefs, and relationship… to the environment.”^5^^(p153)^ Theoretical models of QoL in dementia similarly emphasize the influence of a wide range of psychological, social, environmental, and cultural factors.^6^ Other potential indices of a sense of “comfort, function and contentment with life” are measures of satisfaction with life and subjective well-being. Satisfaction with life entails a global evaluation of one’s current life while subjective well-being reflects the experience of an appropriate balance of positive and negative emotions.^7^ Well-being can be considered as a state of equilibrium or balance which is affected by life events or challenges.^8^ These aspects have been less widely studied in relation to dementia.

A recent systematic review^9^ indicates that numerous individual variables show small associations with self-rated QoL when assessed at the same time, whereas only a very few variables emerge as moderately associated. These are primarily social or psychological in nature; in this review, depression was moderately associated with poorer QoL (effect size, −0.31), while being more socially engaged (0.31) and having a positive relationship with one’s carer (0.38) were moderately associated with better QoL. Models combining several individual variables, mainly basic demographic features, symptoms, and comorbidity, account for only a small proportion of the variance in QoL scores.^10^–^12^ The available evidence, therefore, provides limited guidance about influences on QoL or possible directions for improving the experience of living with dementia.

This suggests the need first for a broader perspective on “living well” with dementia that is commensurate with key definitions and theoretical models, and second for a more comprehensive approach to modeling the factors associated with capability to “live well” with dementia. The Improving the experience of Dementia and Enhancing Active Life (IDEAL) cohort study^13^,^14^ has been set up in part to address this need, using a theoretically derived conceptual framework as a basis for examining multiple influences on living well with dementia. In this framework, the potential for living well is influenced by, and reflects the balance between, the unique set of resources that each person brings to the situation and the particular challenges faced. Resources are the person’s accumulated experiences and abilities together with current social capitals, assets, and resources in the socioenvironmental, psychological, economic, and physical domains.^15^,^16^ Challenges are the personal, social, physical, and practical impact of the disability resulting from the development and progression of dementia.^17^ Here, we use data from the IDEAL initial interviews to model the way in which the social, psychological, and physical resources that the person is able to deploy, and the specific challenges encountered during the development and progression of dementia, are associated with perceptions of capability to “live well” with the condition among people with mild-to-moderate dementia living in community settings.

## METHODS

### Design

IDEAL is a longitudinal cohort study involving people with dementia and, where available, their primary carers, recruited through 29 National Health Service (NHS) sites throughout England, Scotland, and Wales. Information is collected through face-to-face interviews conducted in participants’ own homes by trained interviewers. The study is overseen by an involvement group of people with dementia and carers, known as the ALWAYs (Action on Living Well: Asking You) group, that assisted with the design and contributes to understanding of the results. The present analysis is based on cross-sectional data from the first wave of data collection and utilizes version 2.0 of the data set. The IDEAL study was approved by the Wales Research Ethics Committee 5 (reference 13/WA/0405) and the Ethics Committee of the School of Psychology, Bangor University (reference –2014,11684). IDEAL is registered with the UK Clinical Research Network (UKCRN), number 16593.

### Participants

Participants were recruited through NHS memory services and other specialist clinics, and via the online UK Join Dementia Research portal www.joindementiaresearch.nihr.ac.uk/, between July 2014 and August 2016. Inclusion criteria required participants to have a clinical diagnosis of dementia (any subtype), to be in the mild-to-moderate stages as indicated by a Mini-Mental State Examination^18^ score of ≥15, and to be living in the community at the time of enrollment, excluding individuals with terminal illness, inability to provide informed consent, and any known potential for home visits to pose a significant risk to researchers. In total, 3105 people with dementia were approached about participation, of whom 363 were ineligible and 1106 declined. Of the 1636 who consented, 8 subsequently proved ineligible and 81 withdrew. This resulted in a sample of 1547 participants with dementia (a response rate of 57% among eligible people with dementia). The majority of participants (1283, 82.9%) had a family member or other informal carer who agreed to participate in the study, and 1045 (67.6%) lived with the participating carer.

### Measures of Capability to “Live Well”

Living well was defined as comprising subjective perceptions of QoL, satisfaction with life and well-being reported by participants with dementia. QoL was assessed using the 13-item Quality of Life in Alzheimer Disease scale (QoL-AD)^19^,^20^ with responses to each item given on a 4-point scale (1 to 4) and the scores added to provide a total score out of 52; higher scores indicate more positive ratings of QoL. Satisfaction with life was assessed using the 5-item Satisfaction with Life Scale (SwLS).^21^ Items are rated on a 7-point scale (1 to 7) and responses are summed to give a total score out of 35, with higher scores indicating greater satisfaction. Well-being was assessed using the World Health Organization-Five Well-Being Index (WHO-5).^22^ Responses are scored on a 6-point scale (0 to 5), summed to give a total out of 25, and multiplied by 4 to give a score out of 100, with higher scores indicating greater well-being.

### Measures of Resources and Challenges Potentially Associated With “Living Well”

In our framework, derived from definitions of “living well” and theoretical models of QoL in dementia, resources include the domains of psychological characteristics and psychological health (eg, personality, optimism, loneliness, depression), physical fitness and physical health (eg, exercise, diet, eyesight), social capitals, assets, and resources (eg, education, income, cultural capital, social networks), and social location (perceptions of one’s place in society, eg, social class, social status). Challenges include the symptoms of dementia and their effects, and the impact of these on ability to manage everyday life with dementia (eg, cognition, functional ability). See Supplementary Table 1 (Supplemental Digital Content 1, http://links.lww.com/WAD/A210) for details of the variables considered in each domain and how these were measured. All data were based on self-report.

### Statistical Methods

The analysis was undertaken in a staged approach. Potential variables within each domain were examined in relation to both statistical significance and clinical relevance. Statistical significance was investigated with the Wald test, and the effect size for an unstandardized regression coefficient needed to be >1.5 for QoL-AD or SwLS, and >5 for WHO-5 to be considered clinically relevant.^23^–^25^ Initial analysis was undertaken within each domain against the multivariate outcome (QoL-AD, SwLS, and WHO-5). Factors that were found to be influential in a univariable investigation were included within a multivariable, multivariate investigation. Recoding of variables (from continuous to ordinal groups, or from groups to binary variables) was undertaken to simplify the model, but effect sizes were retained. Multiple imputation by chained equations was conducted to allow for the missing response data. For the complete model, the latent factors representing the 5 domains were regressed on the living well latent factor, adjusting for age, sex, and dementia subtype. The model is parameterized to reflect positive associations indicating enhanced living well outcomes. A coefficient estimate was assumed to be significant if its 95% confidence interval (CI) did not include 0. See the Supplementary Section (Supplemental Digital Content 1, http://links.lww.com/WAD/A210) on statistical methods for expanded details.

## RESULTS

### Participant Characteristics

Characteristics of the 1547 participants and scores on the 3 living well measures are summarized in Table 1. The overall mean scores and SDs were 36.8 (5.9) for QoL-AD, 26.1 (6.1) for SwLS, and 60.9 (20.6) for WHO-5. There were no differences according to sex, but mean ratings were lower for younger people and those with Parkinson disease dementia and dementia with Lewy bodies.

**TABLE 1:**
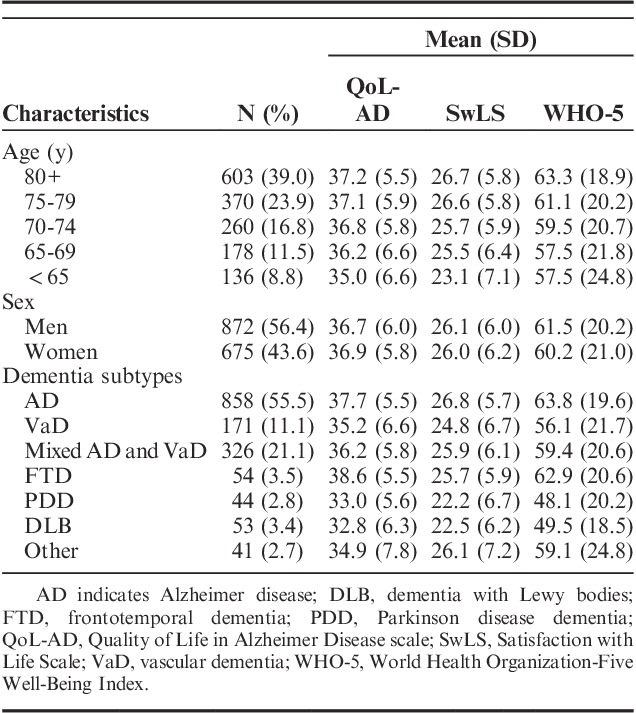
Scores on Measures of Living Well by Age, Sex, and Dementia Subtype

### Variables Included in the Analysis

The variables selected for each domain through univariable multivariate modeling are summarized in Table 2. Full details of the stages of modeling are given in Supplementary Table 2 (Supplemental Digital Content 1, http://links.lww.com/WAD/A210).

**TABLE 2:**
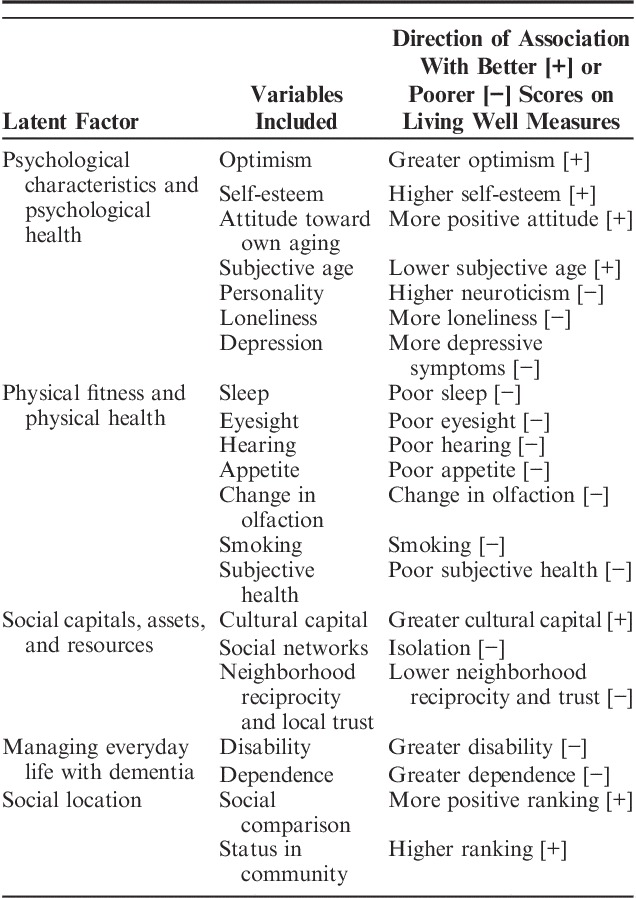
Variables Included in the 5 Latent Factors and Direction of Effect

### Relationships Among the Latent Variables

**FIGURE 1:**
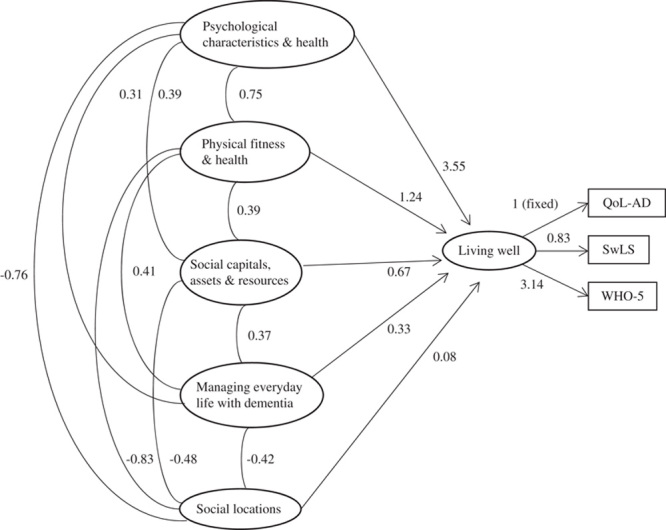
Complete model using imputed data and adjusting for age, sex, and dementia subtype (n=1547). Direction of scoring: lower scores for living well reflect better ability to live well, lower scores for psychological characteristics and psychological health, physical fitness and physical health, social capitals, assets, and resources, and managing everyday life with dementia reflect better experiences or functioning in those domains, and higher scores for social location reflect higher ratings of perceived social status. QoL-AD indicates Quality of Life in Alzheimer Disease scale; SwLS, Satisfaction with Life Scale; WHO-5, World Health Organization-Five Well-Being Index.

The relationship between each of the domains and the living well latent is presented in Figure 1, and further detail is provided in Supplementary Table 3 (Supplemental Digital Content 1, http://links.lww.com/WAD/A210), including correlations between domains. Individual associations with living well were 4.86 (95% CI: 4.54-5.18) for the psychological characteristics and psychological health domain, −4.66 (95% CI: −5.72 to −3.60) for social location, 4.21 (95% CI: 3.84-4.58) for physical fitness and physical health, 2.83 (95% CI: 2.23-3.44) for social capitals, assets, and resources, and 1.98 (95% CI: 1.61-2.35) for managing everyday life with dementia. Following multiple imputation analysis and with adjustment, the model shows that the psychological characteristics and psychological health domain was most strongly associated with living well (3.55; 95% CI: 2.93-4.17). Effect sizes for the other domains ranged from 1.23 to 0.08 (physical fitness and physical health: 1.23, 95% CI: −0.01 to 2.58; social capitals, assets, and resources: 0.67; 95% CI: −0.04 to 1.38; managing everyday life with dementia: 0.33; 95% CI: −0.06 to 0.71; social location: 0.08; 95% CI: −2.10 to 2.26). These factors did not have independent associations with living well when included alongside the psychological characteristics and psychological health domain. Examination of correlations between the latent factors for the 5 domains shows particularly strong associations between the psychological and physical domains and social location (>0.7).

### Impact of Changes for Scores on Living Well Measures

These effects from the standardized analysis were converted back to show the associated change in scores on the outcome variables. For each unit increase in the latent score for each domain, we present the associated changes in scores on the living well measures. These results, seen in Table 3, show that a 1 unit increase in psychological characteristics and psychological health was associated with an increase of 3.55 (95% CI: 2.93-4.17) points on QoL-AD, 2.94 (95% CI: 2.40-3.49) points on SwLS and 11.14 (95% CI: 9.14-13.15) points on WHO-5. A 1 unit increase in the physical fitness and physical health latent factor was associated with a ∼1 point increase in QoL-AD and SwLS and a 3.9 point increase in WHO-5. For the other 3 latent factors, a 1 unit increase was related to a ≤1 point increase on QoL-AD and SwLS and a ≤2 point increase on WHO-5.

**TABLE 3:**
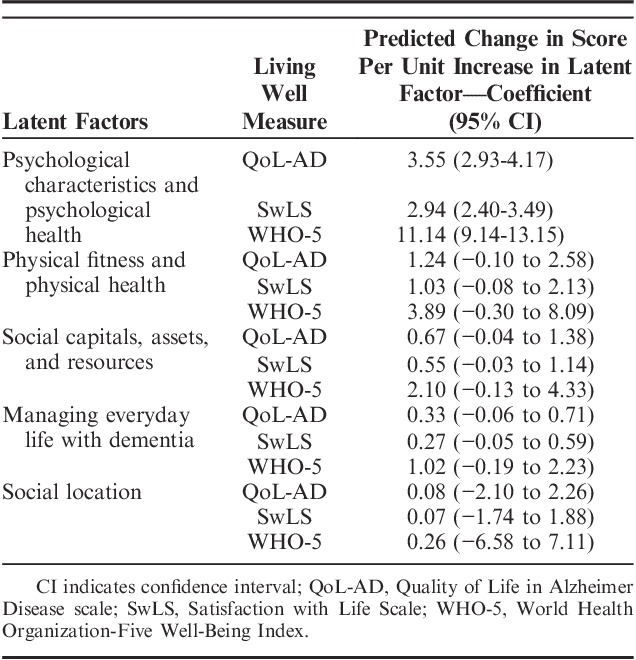
Changes in Scores on Measures of Living Well Per Unit Increase in Each Latent Factor, Adjusted for Age, Sex, and Dementia Subtype and All Latent Factors, With Multiple Imputations; Domains are Ranked According to Size of Predicted Change

## DISCUSSION

Using data from 1547 people living with mild-to-moderate dementia participating in the IDEAL study, we have presented a comprehensive model of factors associated with perceived ability to live well with the condition, conceptualized as balancing resources and challenges. IDEAL is one of very few large studies to explore subjective perceptions of ability to live well with dementia among people in the mild-to-moderate stages of the condition living in the community, both with or without the support of a carer. It is unique in combining the constructs of QoL, satisfaction with life and well-being to provide a comprehensive measure of living well, in the wide range of personal, psychological, physical, social, and environmental factors examined, and in drawing a study population from numerous socially and environmentally diverse areas in Great Britain. The model presented here shows that, when domains are considered individually, the domain of psychological characteristics and psychological health is most strongly associated with concurrent perceptions of living well, followed by social locations and physical fitness and physical health; relatively smaller effect sizes were observed for the domains of social capitals, assets, and resources, and managing everyday life with dementia. When domains are considered together, the psychological domain is dominant in the model. When consulted about the model, the ALWAYs involvement group members thought it was reasonably easy to understand, seemed logical, and provided support for their sense of the important aspects contributing to their ability to live well with the condition.

The psychological characteristics and psychological health domain emerged as particularly important. The dominance of psychological characteristics and psychological health may in part relate to the nature of the constructs being measured, as the self-ratings of psychological features are most similar to the subjective perception of living well when measured at the same time. However, the correlations between domains and living well measures are accounted for in the model and none had perfect correlations; furthermore, this study considered a wide range of factors in the psychological domain such as personality traits, optimism, self-esteem, and attitudes toward own aging, in addition to depression. Evidence from a number of studies indicates that poor psychological health, represented by higher scores on measures of depressive symptoms, is associated with lower ratings of QoL.^9^,^12^,^26^ Although psychological characteristics have traditionally been accorded limited emphasis in studies of QoL in people with dementia, recent work has begun to consider a wider range of psychological variables, including personality traits.^12^

The strengths of our approach lie in the presentation of a detailed model that shows the relative associations of 5 latent factors, reflecting distinct domains, with subjective perceptions of ability to live well with dementia, based on a large sample of people with mild-to-moderate dementia. The modeling included a detailed investigation of and adjustment for missing data via multiple imputations. Missing data were observed within both the measured variables and the outcomes; while low levels of missing data (<3%) were observed for the SwLS and WHO-5, the percentage of missing data for QoL-AD was higher at 9.4%. The modeling process included maximal information, allowing for continuous, ordinal, and binary variables.

There are several limitations to consider. Our investigation estimates the impacts of variables relating to living well at the same timepoint, and hence causal direction cannot be inferred. Although all variables were potentially important, some degree of selection was required in developing the model. Despite the large sample size, some factors did not show statistical significance in the first stage of modeling, or showed significance but were thought less clinically relevant, and the factors remaining within the latent structure were those that showed domain-specific relationships. Hence, some small effect sizes may have been dismissed within the final modeling stage. Some variables assessed in IDEAL were not suitable for inclusion in the structural equation modeling as they did not have linear relationships with living well measures. Some variables were excluded because they were only available for those individuals with a participating carer; for example, ratings of the quality of relationship with the carer. Others were available only through single questions embedded in other measures, and hence less amenable to inclusion; one example in the physical health domain is pain. These variables remain to be explored in future work. Our model is based on self-ratings made by the participants with dementia. While concerns are sometimes raised about the impact of a potential lack of awareness on self-ratings of constructs such as QoL, previous research has shown that variations in awareness are of minor relevance in this regard.^12^ The validity of self-ratings reflecting the subjective perceptions of people living with mild-to-moderate dementia is now widely accepted.

The findings of this study suggest that living well with dementia might be enhanced through improving psychological and physical health as well as addressing other social factors. Although the greatest gain in living well ratings is likely to be achieved through positive increases in factors within the psychological domain, all 5 domains, and all individual factors within the 5 domains, were individually associated with perceived capability to live well with dementia. Although some factors are unlikely to be amenable to intervention, there are several modifiable factors in each domain. For example, while the variables included in the psychological characteristics and psychological health domain encompass some traits, such as dispositional optimism and the personality trait of neuroticism, which may not be direct targets for intervention, other variables such as depression and loneliness may offer more potential for change. Improving physical health where possible, and enabling people to manage disability more effectively, could also improve capability to live well. Social factors that impact on experience in the psychological domain may also provide immediate options for intervention; for example, community efforts to address isolation, enhance neighborhood trust, and increase social engagement could help to address depression and loneliness. Our findings also support the potential for developing an integrated approach to evaluating outcomes that reflect the experiences and needs of people with dementia through creating a new scale measuring “living well” with dementia. In supporting people to “live well” with dementia, our findings reflect the need to take account, not only of disease-related factors, but also of the multiple personal and social factors impacting on psychological health and well-being, as outlined in the recent operationalization of the construct of social health in relation to dementia.^27^ A comprehensive approach to enabling people with dementia, and family carers, to balance resources and challenges must acknowledge this complexity and address multiple factors in an integrated manner.

In conclusion, this study provides new evidence about factors associated with the subjective experience of living well with dementia in the mild-to-moderate stages and about potential targets for immediate intervention. We have adopted a broad perspective on living well and demonstrated that, while in a combined analysis the domain of psychological characteristics and psychological health is most strongly associated with living well, the domains of physical fitness and health, social capitals, assets, and resources, managing everyday life with dementia, and social location all contribute to the overall evaluation of living well when considered individually. Increased understanding of the contribution of these wide-ranging psychological and social factors will help to yield new approaches to enhancing the ability to live well with dementia.

## Supplementary Material

SUPPLEMENTARY MATERIAL

Supplemental Digital Content is available for this article. Direct URL citations appear in the printed text and are provided in the HTML and PDF versions of this article on the journal's website, www.alzheimerjournal.com.
