# Supplementary material for: A Comprehensive Model of Factors Associated With Subjective Perceptions of “Living Well” With Dementia: Findings From the IDEAL Study
Source: Alzheimer Dis Assoc Disord. 2018 Dec 5;33(1):36–41. doi: 10.1097/WAD.0000000000000286 (PMC6416010; doi:10.1097/WAD.0000000000000286)
Supplement: SUPPLEMENTARY MATERIAL [file wad-33-36-s001.docx]

**Supplementary information**

**Statistical methods**

The analysis first investigated the relationships between individual measures and living well outcomes using linear regression modelling and adjusting for age, sex and dementia subtypes. This was used to ensure directions and strengths of individual associations. Based on the five domains in the conceptual model (Supplementary Table 1), all variables identified within the same domain were fitted in one multivariate regression model adjusting for age, sex and subtypes. Based on the adjusted results, three selection criteria were applied to identify the important variables related to living well (Quality of Life in Alzheimer’s Disease scale (QoL-AD),^1, 2^ Satisfaction with Life Scale (SwLS),^3^ World Health Organization-Five Well-Being Index (WHO-5)^4^) and to simplify the model as much as possible:

1. Statistical significance: Wald test was used to examine whether the associations between living well outcomes and a specific measurement achieved statistical significance in the multivariate model.
2. Meaningful difference: The effect sizes were considered to be meaningful when unstandardised regression coefficients achieved QoL-AD>1.5 or SwLS>1.5 or WHO-5>5.0. These cut-offs were determined to balance the need for clinical relevance and based on the literature.^5-7^
3. Binary/ordinal variables: If there was a dose-response relationship, an ordinal model was used for the variable. Categorical variables were regrouped into binary variables if appropriate (given similar effect sizes at different levels).

After the selection process using multivariate modelling, structural equation modelling (SEM) was employed to generate a latent factor for selected variables within each domain and build a structural model examining the associations between individual latent factors and the living well latent with QoL-AD fixed at 1. The variances of individual latent factors were fixed at 1. The results of SEM for the five individual constructs are reported in Supplementary Table 2. A full model was fitted to include all five latent factors and adjusted for age, sex and dementia subtypes. To account for correlations between latent factors and stabilise estimates in the full model, two variables from the psychological characteristics and psychological health domain, attitude toward own ageing and depression, were found to also be important in the managing everyday life with dementia domain. To enable the model to reflect a positive perspective on ‘living well’ the scales of the three living well measures were reversed. The results of the full model are reported in Supplementary Table 3. Quasi-maximum likelihood estimation methods were used to estimate coefficients and appropriate confidence intervals for categorical/ordinal variables.

Multiple imputation (MI) was used to address missing data in selected variables and living well outcomes. A formal MI should impute all individual items within a scale.^8^ However, due to the combination of the complex model and the proportion of missing data, the formal MI approach could not be applied to the full model in a single attempt. To identify a reasonable method balancing efficiency and accuracy, MI was first conducted within each latent factor and missing variables were imputed by i) categories, ii) total scores and iii) individual items, using the method of multiple imputation by chained equations, and for continuous variables predictive mean matching. Age, sex and dementia subtypes were also included in the imputation model. If the SEM results were similar across different imputed datasets, the most efficient method (categories>total scores>individual items) was used for the full model. Estimates from 10 imputed datasets were combined using Rubin’s rules.^8^ Based on the results of imputed full model, changes in QoL-AD, SwLS and WHO-5 scores were estimated for per unit increase in the five latent factors. All analyses were conducted using Stata 14.0.^9^

Supplementary Table 1. Variables considered for inclusion under each domain

| Factor | Measure |
| --- | --- |
| Psychological Characteristics and Psychological Health (PSY) | |
| Personality | Mini-IPIP^10^ |
| Religious belief | Single item^11^ |
| Spirituality | Single item |
| Optimism | Life Orientation Test-Revised^12^ |
| Self-esteem | Rosenberg Self-Esteem Scale^13^; single item^14^ |
| Self-acceptance | Ryff Scales of Psychological Well-Being^15^ self-acceptance subscale^16^ |
| Self-efficacy | Generalized Self-Efficacy Scale^17^ |
| Continuity of sense of self | Single item |
| Loneliness | De Jong Gierveld Loneliness Scale^18^; single item |
| Depression | Geriatric Depression Scale-10^19^ |
| Stressful life events | Social Readjustment Rating Scale^20^ abbreviated 10-item version |
| Attitudes towards own ageing | Philadelphia Geriatric Center Morale Scale^21^ |
| Subjective age | Single item |
| Experience of stigma | Stigma Impact Scale^22, 23^ abbreviated 4-item version |
| Physical Fitness and Health (PHY) | |
| Physical activity | General Practice Physical Activity Questionnaire^24^ |
| Smoking | Current smoker/former smoker/never smoked |
| Alcohol consumption | Currently does/does not consume alcohol |
| Appetite | Short Nutritional Assessment Questionnaire^25^ |
| Eyesight | Single item^16^ |
| Hearing | Single item^16^ |
| Change in gustation | Single item^26^ |
| Change in olfaction | Single item^26^ |
| Sleep quality | Single item |
| Falls | Number of falls in past year^16^ |
| Co-morbid conditions | Charlson Co-morbidity Index^27, 28^ |
| Self-rated health | Single item^29^ |
| Social capitals, assets and resources (CAR) | |
| Education | Highest level of education achieved |
| Income | Income adjusted for household size^30^ |
| Social capital | Resource Generator-UK^31^ |
| Cultural capital | Cultural Capital and Social Exclusion Survey^32^ |
| Social network | Lubben Social Network Scale^33^ |
| Personal relations | Office for National Statistics Social Capital Scale^34^ |
| Reciprocity and local trust | Office for National Statistics Social Capital Scale^34^ |
| Social participation | Office for National Statistics Social Capital Scale^34^ |
| Civic participation | Office for National Statistics Social Capital Scale^34^ |
| Managing Everyday Life with Dementia (MEL) | |
| Cognition | Addenbrooke’s Cognitive Examination-III^35^ |
| Functional ability | Functional Assessment Questionnaire amended 11-item version^36, 37^ |
| Dependence | Dependence Scale^38^ |
| Social Location (SLC) | |
| Social class | Socio-economic status based on occupation^39^ |
| Social comparison | Single item |
| Social status | MacArthur Scale of Subjective Social Status (social ladder ^40^ |
| Community status | MacArthur Scale of Subjective Social Status (community ladder ^40^ |

Supplementary Table 2. Results of structural equation modelling for each of the five latent factors

1. Psychological characteristics and psychological health (PSY)

|  | Model 1 | Model 2 |
| --- | --- | --- |
| Measurement: living well |  |  |
| QoL-AD | 1 (fixed) | 1 (fixed) |
| SwLS | 0.87 (0.81, 0.93) | 0.87 (0.81, 0.93) |
| WHO-5 | 3.19 (2.98, 3.40) | 3.18 (2.97, 3.39) |
|  |  |  |
| Measurement: psychological characteristics and health | | |
| Personality neuroticism |  |  |
| Continuous score | 1.76 (1.56, 1.96) | 1.78 (1.58, 1.98) |
| Optimism |  |  |
| Continuous score | -2.01 (-2.23, -1.80) | -2.00 (-2.22, -1.79) |
| Self-esteem |  |  |
| Ordinal variable | -0.41 (-0.46, -0.36) | -0.41 (-0.46, -0.36) |
| Attitude toward own ageing |  |  |
| Continuous score | -1.08 (-1.15, -1.01) | -1.07 (-1.15, -1.00) |
| Depression |  |  |
| Yes vs no (ref) | 0.33 (0.31, 0.34) | 0.33 (0.31, 0.34) |
| Subjective age |  |  |
| Ordinal variable | -0.17 (-0.22, -0.13) | -0.17 (-0.22, -0.13) |
| Loneliness |  |  |
| Yes vs no (ref) | 0.10 (0.08, 0.13) | 0.10 (0.08, 0.13) |
|  |  |  |
| Structural |  |  |
| PSY -> Living well | 4.86 (4.55, 5.17) | 4.86 (4.54, 5.18) |

*Note: Latent variance of psychological characteristics and health fixed at 1; Model 1: unadjusted; Model 2: adjusted for age, sex and subtypes.* *QoL-AD: Quality of Life in Alzheimer’s Disease scale; SwLS: Satisfaction with Life Scale; WHO-5: World Health Organization-Five Well-Being Index.*

(b) Physical fitness and health (PHY)

|  | Model 1 | Model 2 |
| --- | --- | --- |
| Measurement: living well |  |  |
| QoL-AD | 1 (fixed) | 1 (fixed) |
| SwLS | 0.81 (0.75, 0.87) | 0.82 (0.76, 0.88) |
| WHO-5 | 3.18 (2.95, 3.42) | 3.19 (2.96, 3.43) |
|  |  |  |
| Measurement: Physical fitness and health | | |
| Poor sleep |  |  |
| Ordinal variable | 0.50 (0.43, 0.57) | 0.51 (0.44, 0.58) |
| Poor eyesight |  |  |
| Ordinal variable | 0.50 (0.44, 0.56) | 0.50 (0.44, 0.56) |
| Poor hearing |  |  |
| Ordinal variable | 0.36 (0.29, 0.43) | 0.35 (0.28, 0.42) |
| Poor self-rated health |  |  |
| Ordinal variable | 0.69 (0.64, 0.75) | 0.70 (0.64, 0.75) |
| Poor appetite |  |  |
| Binary variable | 0.15 (0.13, 0.18) | 0.15 (0.13, 0.18) |
| Smoking |  |  |
| Ordinal variable | 0.12 (0.08, 0.15) | 0.11 (0.07, 0.15) |
| Change in olfaction |  |  |
| Yes vs No (ref.) | 0.08 (0.06, 0.11) | 0.09 (0.06, 0.11) |
|  |  |  |
| PHY -> Living well | -4.29 (-4.64, -3.94) | -4.21 (-4.58, -3.84) |

*Note: Latent variance of physical fitness and health fixed at 1; Model 1: unadjusted; Model 2: adjusted for age, sex and subtypes.* *QoL-AD: Quality of Life in Alzheimer’s Disease scale; SwLS: Satisfaction with Life Scale; WHO-5: World Health Organization-Five Well-Being Index.*

(c) Social capitals, assets and resources (CAR)

|  | Model 1 | Model 2 |
| --- | --- | --- |
| Measurement: living well |  |  |
| QoL-AD | 1 (fixed) | 1 (fixed) |
| SwLS | 0.84 (0.78, 0.91) | 0.85 (0.78, 0.92) |
| WHO-5 | 3.18 (2.93, 3.43) | 3.17 (2.93, 3.41) |
|  |  |  |
| Measurement: Social capitals, assets and resources | | |
| Social network |  |  |
| Isolated vs not isolated (ref.) | -0.23 (-0.27, -0.18) | -0.22 (-0.26, -0.19) |
| Cultural capital |  |  |
| Ordinal variable | 0.49 (0.40, 0.58) | 0.55 (0.46, 0.64) |
| Reciprocity and local trust |  |  |
| Not likely vs likely* (ref.) | -0.15 (-0.18, -0.11) | -0.13 (-0.16, -0.09) |
|  |  |  |
| Structural |  |  |
| CAR -> Living well | 2.69 (2.12, 3.27) | 2.83 (2.23, 3.44) |

*Note: Latent variance of social capitals, assets and resources fixed at 1; Model 1: unadjusted; Model 2: adjusted for age, sex and subtypes *Likelihood of return of lost wallet. QoL-AD: Quality of Life in Alzheimer’s Disease scale; SwLS: Satisfaction with Life Scale; WHO-5: World Health Organization-Five Well-Being Index.*

(d) Managing everyday life with dementia (MEL)

|  | Model 1 | Model 2 |
| --- | --- | --- |
| Measurement: living well |  |  |
| QoL-AD | 1 (fixed) | 1 (fixed) |
| SwLS | 0.82 (0.75, 0.88) | 0.83 (0.76, 0.89) |
| WHO-5 | 3.16 (2.90, 3.42) | 3.18 (2.93, 3.44) |
|  |  |  |
| Measurement: Managing everyday life | | |
| Functional ability |  |  |
| Ordinal variable | 0.92 (0.83, 1.00) | 0.91 (0.82, 1.00) |
| Dependence |  |  |
| Ordinal variable | 0.89 (0.81, 0.96) | 0.89 (0.81, 0.97) |
|  |  |  |
| MEL -> Living well | -2.23 (-2.60, -1.86) | -1.98 (-2.35, -1.61) |

*Note: Latent variance of managing everyday life with dementia fixed at 1; Model 1: unadjusted; Model 2: adjusted for age, sex and subtypes. QoL-AD: Quality of Life in Alzheimer’s Disease scale; SwLS: Satisfaction with Life Scale; WHO-5: World Health Organization-Five Well-Being Index.*

(e) Social Location (SLC)

|  | Model 1 | Model 2 |
| --- | --- | --- |
| Measurement: living well |  |  |
| QoL-AD | 1 (fixed) | 1 (fixed) |
| SwLS | 0.84 (0.78, 0.91) | 0.84 (0.78, 0.91) |
| WHO-5 | 3.16 (2.92, 3.41) | 3.16 (2.92, 3.41) |
|  |  |  |
| Measurement: Social location |  | |
| Social comparison |  |  |
| Ordinal variable | 0.50 (0.42, 0.59) | 0.54 (0.45, 0.63) |
| Community status |  |  |
| Ordinal variable | 0.31 (0.26, 0.36) | 0.29 (0.24, 0.34) |
|  |  |  |
| Structural |  |  |
| SLC -> Living well | 4.29 (3.64, 4.94) | 4.66 (3.60, 5.72) |

*Note: Latent variance of social location fixed at 1; Model 1: unadjusted; Model 2: adjusted for age, sex and subtypes. QoL-AD: Quality of Life in Alzheimer’s Disease scale; SwLS: Satisfaction with Life Scale; WHO-5: World Health Organization-Five Well-Being Index.*

Supplementary Table 3. Coefficients and confidence intervals for the complete model

|  | Unadjusted | | Adjusted for age, sex and dementia sub-type | |
| --- | --- | --- | --- | --- |
|  | Coef. | (95% CI) | Coef. | (95% CI) |
| Measurement model - living well | |  | |  |
| QoL-AD | 1 | (fixed) | 1 | (fixed) |
| SwLS | 0.83 | (0.78, 0.88) | 0.83 | (0.78, 0.88) |
| WHO-5 | 3.15 | (2.97, 3.33) | 3.14 | (2.96, 3.32) |
| Structural associations | |  | |  |
| PSY | 3.49 | (2.93, 4.05) | 3.55 | (2.93, 4.17) |
| SLC | -0.06 | (-1.34, 1.21) | 0.08 | (-2.10, 2.26) |
| MEL | 0.33 | (-0.06, 0.73) | 0.33 | (-0.06, 0.71) |
| PHY | 1.23 | (0.36, 2.11) | 1.23 | (-0.10, 2.58) |
| CAR | 0.58 | (0.13, 1.03) | 0.67 | (-0.04, 1.38) |
| Correlations between latent factors | |  | |  |
| PSY, SLC | -0.77 | (-0.88, -0.66) | -0.76 | (-0.87, -0.65) |
| PSY, MEL | 0.32 | (0.24, 0.41) | 0.31 | (0.23, 0.40) |
| PSY, PHY | 0.75 | (0.70, 0.80) | 0.75 | (0.70, 0.80) |
| PSY, CAR | 0.41 | (0.31, 0.52) | 0.39 | (0.29, 0.50) |
| SLC, MEL | -0.43 | (-0.53, -0.33) | -0.42 | (-0.52, -0.32) |
| SLC, PHY | -0.84 | (-0.95, -0.73) | -0.83 | (-0.94, -0.72) |
| SLC, CAR | -0.52 | (-0.68, -0.36) | -0.48 | (-0.64, -0.32) |
| MEL, PHY | 0.41 | (0.34, 0.47) | 0.41 | (0.35, 0.47) |
| MEL, CAR | 0.38 | (0.27, 0.48) | 0.37 | (0.27, 0.47) |
| PHY, CAR | 0.40 | (0.30, 0.50) | 0.39 | (0.29, 0.48) |

*Note: QoL-AD: Quality of Life in Alzheimer’s Disease scale; SwLS: Satisfaction with Life Scale; WHO-5: World Health Organization-Five Well-Being Index; SLC: Social Location; CAR: Social capitals, assets and resources; PSY: Psychological Characteristics and Psychological Health; PHY: Physical Fitness and Health; MEL: Managing Everyday Life with Dementia*

**References**

1. Logsdon RG, Gibbons LE, McCurry SM, et al. Quality of life in Alzheimer's disease: patient and caregiver reports. In: Albert SM, Logsdon RG, editors. *Assessing quality of life in dementia*. New York: Springer; 2000. p. 17-30.

2. Logsdon RG, Gibbons LE, McCurry SM, et al. Assessing quality of life in older adults with cognitive impairment. *Psychosom Med*. 2002;64:510-519.

3. Diener E, Emmons RA, Larsen RJ, et al. The Satisfaction With Life Scale. *J Pers Assess*. 1985;49:71-75.

4. Bech P. Measuring the dimension of psychological general well-being by the WHO-5. *Qual Life Newslett*. 2004;32:15-16.

5. Clare L, Woods RT, Nelis SM, et al. Trajectories of quality of life in early-stage dementia: individual variations and predictors of change. *Int J Geriatr Psychiatry*. 2014;29:616-623.

6. Kobau R, Sniezek J, Zack MM, et al. Well-being assessment: an evaluation of well-being scales for public health and population estimates of well-being among US adults. *Appl Psychol Health Well-Being*. 2010;2:272-297.

7. Topp CW, Østergaard SD, Søndergaard S, et al. The WHO-5 Well-Being Index: a systematic review of the literature. *Psychother Psychosom*. 2015;84:167-176.

8. Rubin DB. Multiple imputation after 18+ years. *J Am Stat Assoc*. 1996;91:473-489.

9. StataCorp. *Stata Multiple-Imputation Reference Manual Release 13*. College Station, TX: StataCorp; 2013.

10. Donnellan MB, Oswald FL, Baird BM, et al. The Mini-IPIP scales: tiny-yet-effective measures of the Big Five factors of personality. *Psychol Assess*. 2006;18:192-203.

11. Loewenthal KM, MacLeod AK, Cinnirella M. Are women more religious than men? Gender differences in religious activity among different religious groups in the UK. *Pers Individ Dif*. 2002;32:133-139.

12. Scheier MF, Carver CS, Bridges MW. Distinguishing optimism from neuroticism (and trait anxiety, self-mastery, and self-esteem): a reevaluation of the Life Orientation Test. *J Pers Soc Psychol*. 1994;67:1063-1078.

13. Rosenberg M. *Society and the adolescent self-image*. Princeton, NJ: Princeton University Press; 1965.

14. Robins RW, Hendin HM, Trzesniewski KH. Measuring global self-esteem: construct validation of a single-item measure and the Rosenberg self-esteem scale. *Pers Soc Psychol Bull*. 2001;27:151-161.

15. Ryff CD, Keyes CL. The structure of psychological well-being revisited. *J Pers Soc Psychol*. 1995;69:719-727.

16. Marmot M, Oldfield Z, Clemens S, et al. English Longitudinal Study of Ageing: Wave 2 2004-2005. In: Service UD, editor. 27th ed. 2017.

17. Schwarzer R, Jerusalem M. Generalized Self-Efficacy Scale. In: Weinman J, Wright S, Johnston M, editors. *Measures in health psychology: a user’s portfolio Causal and control beliefs*. Windsor, UK: NFER-NELSON; 1995. p. 35-37.

18. De Jong Gierveld J, Tilburg TV. A 6-item scale for overall, emotional, and social loneliness confirmatory tests on survey data. *Res Aging*. 2006;28:582-598.

19. Almeida OP, Almeida SA. Short versions of the Geriatric Depression Scale: a study of their validity for the diagnosis of a major depressive episode according to ICD-10 and DSM-IV. *Int J Geriatr Psychiatry*. 1999;14:858-865.

20. Holmes TH, Rahe RH. The Social Readjustment Rating Scale. *J Psychosom Res*. 1967;11:213-218.

21. Lawton MP. The Philadelphia Geriatric Center Morale Scale: a revision. *J Gerontol*. 1975;30:85-89.

22. Burgener SC, Berger B. Measuring perceived stigma in persons with progressive neurological disease: Alzheimer's dementia and Parkinson's disease. *Dementia*. 2008;7:31-53.

23. Fife BL, Wright ER. The dimensionality of stigma: a comparison of its impact on the self of persons with HIV/AIDS and cancer. *J Health Soc Behav*. 2000;41:50-67.

24. National Health Service. *The General Practice Physical Activity Questionnaire (GPPAQ): a screening tool to assess adult physical activity levels, within primary care*. London: Department of Health; 2009.

25. Wilson MM, Thomas DR, Rubenstein LZ, et al. Appetite assessment: simple appetite questionnaire predicts weight loss in community-dwelling adults and nursing home residents. *Am J Clin Nutr*. 2005;82:1074-1081.

26. Heald AE, Pieper CF, Schiffman SS. Taste and smell complaints in HIV-infected patients. *AIDS*. 1998;12:1667-1674.

27. Charlson ME, Charlson RE, Peterson JC, et al. The Charlson comorbidity index is adapted to predict costs of chronic disease in primary care patients. *J Clin Epidemiol*. 2008;61:1234-1240.

28. Charlson ME, Pompei P, Ales KL, et al. A new method of classifying prognostic comorbidity in longitudinal studies: development and validation. *J Chronic Dis*. 1987;40:373-383.

29. Bowling A. Just one question: if one question works, why ask several? *J Epidemiol Community Health*. 2005;59:342-345.

30. Bridges S, Doyle M, Fuller E, et al. *Health Survey for England, 2012. Volume 2: Methods and Documentation*. London: Health and Social Care Information Centre; 2013.

31. Webber MP, Huxley PJ. Measuring access to social capital: the validity and reliability of the Resource Generator-UK and its association with common mental disorder. *Soc Sci Med*. 2007;65:481-492.

32. Thomson K. *Cultural capital and social exclusion survey: technical report*. London: National Centre for Social Research; 2004.

33. Lubben J, Blozik E, Gillmann G, et al. Performance of an abbreviated version of the Lubben Social Network Scale among three European community-dwelling older adult populations. *Gerontologist*. 2006;46:503-513.

34. Office for National Statistics. *Harmonised concepts and questions for social data sources, secondary standards. Social capital*. Titchfield, UK: Office for National Statistics; 2008.

35. Hsieh S, Schubert S, Hoon C, et al. Validation of the Addenbrooke's Cognitive Examination III in frontotemporal dementia and Alzheimer's disease. *Dement Geriatr Cogn Disord*. 2013;36:242-250.

36. Martyr A, Clare L, Nelis SM, et al. Verbal fluency and awareness of functional deficits in early-stage dementia. *Clin Neuropsychol*. 2012;26:501-519.

37. Pfeffer RI, Kurosaki TT, Harrah CH, Jr., et al. Measurement of functional activities in older adults in the community. *J Gerontol*. 1982;37:323-329.

38. Brickman AM, Riba A, Bell K, et al. Longitudinal assessment of patient dependence in Alzheimer disease. *Arch Neurol*. 2002;59:1304-1308.

39. Office for National Statistics. *Standard Occupational Classification 2010. Volume 3. The National Statistics Socio-economic Classification: (Rebased on the SOC2010) User Manual*. Basingstoke: Palgrave Macmillan; 2010.

40. Adler NE, Epel ES, Castellazzo G, et al. Relationship of subjective and objective social status with psychological and physiological functioning: preliminary data in healthy white women. *Health Psychol*. 2000;19:586-592.
